# Complete Characterization of Degradation Byproducts of Bemotrizinol and Degradation Pathway Associated with Sodium Hypochlorite Treatment

**DOI:** 10.3390/molecules30142935

**Published:** 2025-07-11

**Authors:** Armando Zarrelli

**Affiliations:** Department of Chemical Sciences, University of Naples Federico II, Via Vicinale Cupa Cintia 26, 80126 Naples, Italy; armando.zarrelli@unina.it; Tel.: +39-081-674472

**Keywords:** bemotrizinol, chlorination, hypochlorite, degradation byproducts, water treatment, degradation mechanism

## Abstract

The aim of this study was to elucidate all the degradation byproducts (DBPs) of bemotrizinol (BEMT) that are associated with sodium hypochlorite treatment. BEMT is a UV filter that is found not only in many personal care products, such as sunscreen and cosmetics, but also as an additive in plastics or clothing to protect them from damage that results from absorbed radiation. BEMT has been detected in wastewater, surface water, and some lake sediments, in quantities from a few ng/L to hundreds of ng/L, to such an extent that, today, it is considered an emerging pollutant. In this study, the UV filter was subjected to oxidation with sodium hypochlorite, which is an oxidant at the base of the disinfection process that is used in most wastewater treatment plants or in swimming pools. Using different chromatographic methods (CC, TLC, HPLC, and GC), the resulting DBP mixture was separated into its main components, which were then identified using one- and two-dimensional nuclear magnetic resonance (NMR) spectroscopy and mass spectrometry. Nineteen DBPs were isolated, and a plausible reaction mechanism was proposed to explain how they were obtained.

## 1. Introduction

Increasing human activities have a continuous impact on aquatic ecosystems through the release of numerous chemicals that may pose a significant threat to habitats [1,2]. Among them, UV filters are potential emerging pollutants that have attracted public interest in the last decade [3,4,5,6]. In accordance with the EC Cosmetics Regulation [7], which applies to the entire EU market, UV filters are defined as substances that are intended exclusively or primarily to protect the skin against a certain range of UV radiation (280–400 nm) by absorbing, reflecting, or scattering it. In this respect, a distinction is made between soluble organic UV filters and mineral- and/or organic pigment-based UV filters [8]. While soluble organic UV filters absorb UV radiation, convert it into heat, and form an invisible protective shield in the upper layer of the skin, pigment-based UV filters reflect, scatter, and absorb solar radiation already at the skin surface. Organic UV filters are distinguished by the position of the absorption zone and their photostability, solubility, and penetration capacity, among other aspects. Therefore, these chemicals are found not only in many personal care products, such as sunscreen and cosmetics, but also as additives in plastics or clothing to protect them from damage from absorbed radiation [9]. UV filters minimize the unwanted effects of UV radiation on the skin, helping to prevent skin aging and skin cancer.

UV filters enter the aquatic ecosystem directly through skin washing, especially during summer activities, or indirectly through wastewater [9,10,11]. Currently, about 60 different filters are available on the global market [12], and it is estimated that tourism in coastal areas results in the release of about 14,000 tons of sunscreen into the oceans every year [13,14]. Not surprisingly, numerous studies have reported their presence in different ecosystems [15,16,17,18].

Some of the effects of sunscreens on marine ecosystems include the following: (*i*) coral bleaching and cell death, compromising their health and survival. In fact, several UV filters are banned in countries such as Palau, Hawaii, Key West, the United States Virgin Islands, and nature reserves in Mexico [19,20,21]. (*ii*) hormonal disruption—some sunscreens can act as endocrine disruptors, causing hormonal imbalances and reproductive changes in several marine organisms. They can even change the sex of some fish species, causing their extinction [22,23]. (*iii*) Death of marine organisms—in some cases, sunscreens are highly toxic to corals, fish, and other marine organisms, causing their death [24,25,26]. (*iv*) blocking photosynthesis—sunscreens can form a film on the surface of the water, limiting the access of light and preventing photosynthesis, with consequences for the health of marine ecosystems [27,28].

**Figure 1:**
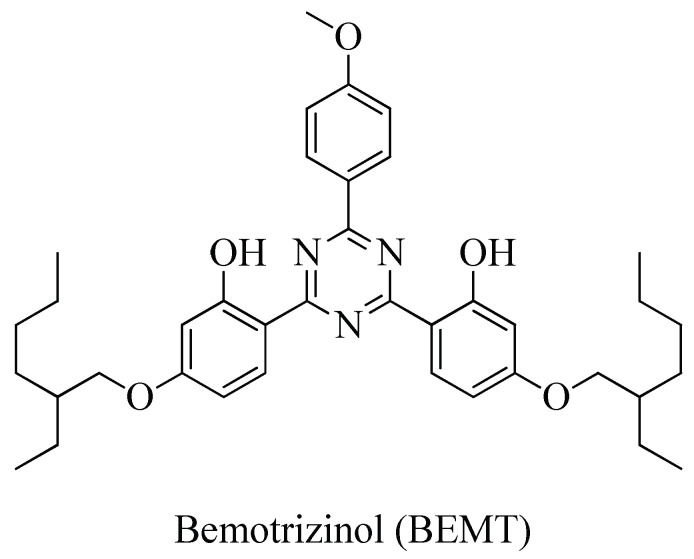
Chemical structure of bemotrizinol.

Bis-ethylhexyloxyphenol methoxyphenyl triazine/2,2′-[6-(4-methoxyphenyl)-1,3,5- triazin-2,4-diyl] bis-[5-[(2-ethylhexyl)oxy] phenol, better known as bemotrizinol (BEMT, Figure 1), is a broadband filter that protects the skin against both UV-B (280–320 nm) and UV-A (320–400 nm) radiation, with the highest protection at 310 nm and 345 nm.

Recent advances in synthetic chemistry have revolutionized the production of bemotrizinol. Researchers have developed a simplified one-pot process, taking advantage of zinc-mediated Negishi coupling and optimized catalyst systems [29]. This innovation not only simplifies the production steps but also improves the purity of the product, reaching over 99%, a significant jump compared to previous methods. Approved in the EU, Asia, Latin America, and Australia, its use is permitted at 10% in the EU, Asia, and Latin America and up to 3% in Japan; however, it is banned in the United States [30].

Bemotrizinol is characterized by a logP of 9.29 [31], a particularly low value that justifies a very low solubility in sea water (< 2 × 10^−4^ ng/L) and even less in pure water (3 × 10^−5^ ng/L) and drinking water (1.5 × 10^−10^ ng/L). BEMT has been detected at concentrations of just over 18 ng/L in surface waters, up to 115 ng/g in some lake sediments, and over 5625 ng/g in water surface microlayers [31]. In some stretches of sea along the French coast overlooking the Mediterranean, concentrations ranging between 2.1 and 14.0 mg/L have been detected in the summer months [32]. BEMT has also been detected in municipal sludge samples from 30 provinces and municipalities across China, at concentrations of up to 300 ng/g [33]. There are few studies evaluating the ecotoxicological profile of this sunscreen, with some measuring its effect on the brine shrimp *Artemia salina* and the marine microalgae *Tetraselmis* [34] and the stress it causes in corals exposed to its presence [35].

**Figure 2:**
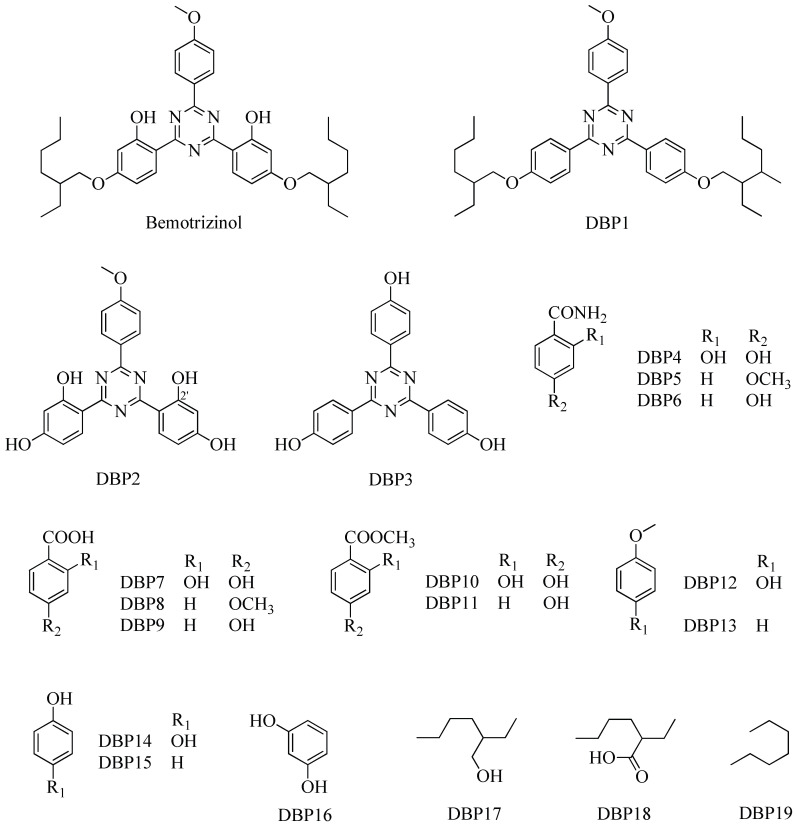
Chemical structures of bemotrizinol and its degradation byproducts DBP1–DBP19.

The aim of this work is to investigate whether BEMT can be degraded, completely or partially, during disinfection processes of wastewater or recreational water. Therefore, this UV filter was subjected to oxidation with sodium hypochlorite [36], the most widely used disinfectant agent in municipal wastewater treatment plants and in swimming pools. The resulting mixture of degradation products was then separated into its main components using different methods, such as column chromatography (CC), thin-layer chromatography (TLC), high-performance liquid chromatography (HPLC), and gas chromatography (GC). The different fractions were then identified and characterized using spectroscopic and spectrometric techniques, primarily via one- and two-dimensional nuclear magnetic resonance (NMR) spectroscopy. Nineteen degradation byproducts (DBPs, Figure 2) of bemotrizinol were isolated, and a plausible reaction mechanism is proposed, which could be useful in predicting the possible DBPs obtainable from structurally related products.

## 2. Results and Discussion

### 2.1. Degradation Experiments 

**Figure 3:**
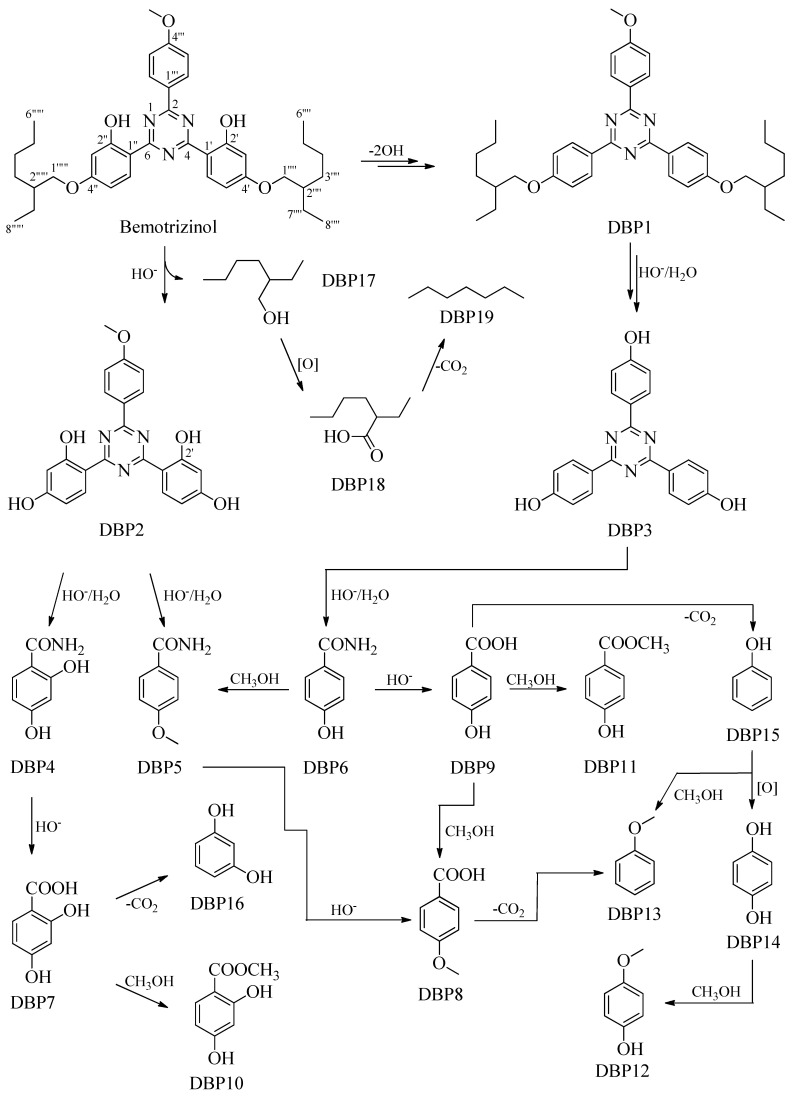
Plausible mechanism for the formation of DBP1-DBP19.

As described in Section 3.2.2., BEMT degradation experiments were performed at two different concentrations: under analytical conditions, at about 10^−5^ M, a concentration slightly higher than that detected in wastewater [32], and, under preparative conditions, at a concentration at least 100 times higher to identify and isolate the DBPs showing the same retention times in the two different conditions. The reaction, followed at regular time intervals of 15 min, was stopped after about 2 h, the time necessary to obtain DBPs in the highest possible quantities, facilitating their isolation and structural identification. Degradation was monitored using HPLC and GC; the obtained DBPs were isolated using column chromatography and HPLC with a purity of at least 95% and then fully characterized using NMR and MS analyses. DBP1–DBP19 (Figure 2) were isolated at relative percentages of 43.98, 0.20, 0.20, 0.20, 0.20, 0.31, 0.41, 0.20, 0.31, 0.10, 0.20, 0.16, 0.10, 0.20, 0.31, 0.10, 1.33, 0.14, and 0.22, respectively, of which DBP1 was described using NMR for the first time. Finally, Figure 3 shows the proposed mechanism for the formation of all DBPs, starting from BEMT. 

### 2.2. Proposed Mechanism for the Formation of Degradation Byproducts DBP1–DBP19

Bemotrizinol, or *bis*-Ethylhexyloxyphenol methoxyphenyl triazine, is a chemical compound based on 1,3,5-triazine, with a methoxyphenyl group linked to the C-2 carbon and two phenolic groups, which are linked to two 2-ethylhexyl chains, which are linked to the C-4 and C-6 carbons (Figure 3). Hydrolysis or, in any case, cleavage of the bond between the phenolic oxygen and the C-1”” carbon leads to the formation of DBP2 and 2-ethylhexan-1-ol (DBP17). The oxidation of the latter results in the corresponding acid DBP18, the decarboxylation of which results in DBP19. DBP2 undergoes degradation of the triazine ring, the opening of which results in the dihydroxylated benzamide DBP4 and the simpler 4-methoxybenzamide DBP5. Of the two amides, the first undergoes hydrolysis and leads to the corresponding acid DBP7, from which the ester DBP10 is obtained via methylation and the resorcinol DBP16 is obtained via decarboxylation. From the 4-methoxybenzamide DBP5, the corresponding acid DBP8 is obtained via saponification, which provides the anisole DBP13 via decarboxylation.

The starting product, with the loss of the hydroxyl functions linked to the C-2′ and C-2” carbons, provides DBP1, which, with the loss of the side chains linked to the C-1”” and C-1””’ carbons, provides DBP3. This, with the opening of the triazine ring, leads to the formation of 4-hydroxybenzamide, DBP6. The latter provides the DBP5 via methylation and the corresponding acid DBP9 via saponification. The decarboxylation of *p*-hydroxybenzoic acid results in the phenol DBP15, and the methylation of the carboxylic function results in the ester DBP11. Finally, the phenol easily explains how the dihydroxybenzene DBP14 and the anisole DBP13 are obtained, while the methylation of one of the two hydroxyl functions of DBP14 results in DBP12.

The lowest-molecular-weight compounds, such as DBP4-DBP19, are aromatic compounds C_6_H_5_C_1_ and/or C_6_H_5_C_0_ or alcohols, acids, and alkanes with a few carbon atoms, less than 6, and are very common degradation byproducts of many organic micropollutants. Generally, however, they are considered intermediates of the oxidative process before the complete mineralization of the starting products is achieved.

### 2.3. Spectral Data

Bemotrizinol (BEMT): 6,6′-(6-(4-Methoxyphenyl)-1,3,5-triazine-2,4-diyl)bis(3-((2- ethylhexyl)oxy) phenol). Gray powder. ^1^H-NMR (CDCl_3_, 400 MHz, room temperature): δ = 8.29 (4H, br s, H-6′ and H-6”, H-2′” and H-6′”), 6.99 (2H, d, J = 8.9, H-3”’ and H-5′”), 6.53 (2H, dd, J = 8.9 and 2.2, H-5′ and H-5”), 6.47 (2H, d, J = 2.2, H-3′ and H-3”), 3.90 (4H, d, J = 6.1, H-1”” and H-1””’), 3.89 (3H, s, OCH_3_), 1.78 (2H, q, J = 6.0, H-2”” and H-2””’), 1.50 (12H, m, H-3””, H-4””, H-7””, H-3””’, H-4””’, and H-7””’), 1.36 (4H, m, H-5”” and H-5””’), 0.98 (6H, t, J = 7.2, H-6”” and H-6””’), 0.93 (6H, t, J = 7.2, H-8”” and H-8””’). ^13^C-NMR (CDCl_3_, 400 MHz, room temperature): δ = 166.89 (C-2, C-4 and C-6), 111.41 (C-1′ and C-1”), 165.25 (C-2′ and C-2”), 103.15 (C-3′ and C-3”), 165.95 (C-4′ and C-4”), 109.85 (C-5′ and C-5”), 132.01 (C-6′ and C-6”), 132.09 (C-1”’), 132.01 (C-2”’ and C-6”’), 115.70 (C-3”’ and C-5”’), 165.95 (C-4”’), 72.22 (C-1”” and C-1””’), 40.63 (C-2”” and C-2””’), 31.86* (C-3”” and C-3””’), 30.47* (C-4”” and C-4””’), 24.40 (C-5”” and C-5””’), 15.48 (C-6”” and C-6””’), 25.20 (C-7”” and C-7””’), 12.50 (C-8”” and C-8””’), 56.88 (OCH_3_). MS-TOF (positive ions): m/z calculated for C_38_H_49_N_3_O_5_ 627.37 [M]^+^; found 628.83 [M + H]^+^ (36 %), 629.83 [M + 1+ H]^+^ (13 %). UV (CH_3_CN) λ_max_/nm 340 (ε/dm^3^ mol^−1^ cm^−1^, 36,000), 309 (31,000).

DBP1: 2,4-bis(4-((2-Ethylhexyl)oxy)phenyl)-6-(4-methoxyphenyl)-1,3,5-triazine. Gray powder. ^1^H-NMR (CDCl_3_, 400 MHz, room temperature): δ = 8.28 (4H, br s, H-2′, H-2”, H-6′ and H-6”), 8.19 (2H, d, J = 8.8, H-2”’ and 6”’), 6.96 (6H, d, J = 8.8, H-3′, H-3”, H-6′, H-6”, H-3”’ and H-5”’), 4.04 (4H, d, J = 5.6, H-1”” and H-1””’), 3.94 (3H, s, OCH_3_), 1.83 (2H, m, H-2”” and H-2””’), 1.53* (4H, m, H-3”” and 3””’), 1.47 (4H, m, H-7”” and H-8””’), 1.41* (4H, m, H-4”” and H-4””’), 1.39 (4H, m, H-5”” and H-5””’), 1.03 (6H, t, J = 7.2, H-8”” and H-8””’), 0.95 (6H, t, J = 7.2, H-6”” and H-6””’). ^13^C-NMR (CDCl_3_, 400 MHz, room temperature): δ = 167.80 (C-2), 169.32 (C-4 and C-6), 124.45 (C-1′, C-1” and C-1”’), 120.05 (C-2′, C-2”, C-6′ and C-6”), 113.46 (C-3′, C-3”, C-5′ and C-5”), 157.65 (C-4′ and C-4”), 131.01 (C-2”’ and C-6”’), 114.78 (C-3”’ and C-5”’), 164.94 (C-4”’), 76.19 (C-1”” and C-1””’), 40.63 (C-2”” and C-2””’), 30.16 (C-3”” and C-3””’), 29.16 (C-4”” and C-4””’), 23.06 (C-5”” and C-5””’), 14.14 (C-6”” and C-6””’), 23.60 (C-7”” and C-7””’), 11.24 (C-8”” and C-8””’), 55.67 (OCH_3_). MS-TOF (positive ions): m/z calculated for C_38_H_49_N_3_O_3_ 595.38 [M]^+^; found 596.82 [M + H]^+^ (30 %), 597.83 [M + 1+ H]^+^ (11 %). UV (CH_3_CN) λ_max_/nm 322 (ε/dm^3^ mol^−1^ cm^−1^, 2,370), 226 (2,200).

DBP2: 4,4′-(6-(4-Methoxyphenyl)-1,3,5-triazine-2,4-diyl)bis(benzene-1,3-diol). Oily liquid. The compound was identified by comparison with an authentic, commercially available sample.

DBP3: 4,4′,4″-(1,3,5-Triazine-2,4,6-triyl)triphenol. Oily liquid. The compound was identified by comparison with an authentic, commercially available sample.

DBP4: 2,4-Dihydroxybenzamide. Gray powder. The compound was identified by comparison with an authentic, commercially available sample.

DBP5: 4-Methoxybenzamide. White powder. The compound was identified by comparison with an authentic, commercially available sample.

DBP6: 4-Hydroxybenzamide. White powder. The compound was identified by comparison with an authentic, commercially available sample.

DBP7: 2,4-Dihydroxybenzoic acid. White powder. The compound was identified by comparison with an authentic, commercially available sample.

DBP8: 4-Methoxybenzoic acid. White powder. The compound was identified by comparison with an authentic, commercially available sample.

DBP9: 4-Hydroxybenzoic acid. White powder. The compound was identified by comparison with an authentic, commercially available sample.

DBP10: Methyl 2,4-dihydroxybenzoate. White powder. The compound was identified by comparison with an authentic, commercially available sample.

DBP11: Methyl 4-hydroxybenzoate. Oily liquid. The compound was identified by comparison with an authentic, commercially available sample.

DBP12: 4-Methoxyphenol. Gray powder. The compound was identified by comparison with an authentic, commercially available sample.

DBP13: Anisole. Straw-colored liquid. The compound was identified by comparison with an authentic, commercially available sample.

DBP14: Hydroquinone. White powder. The compound was identified by comparison with an authentic, commercially available sample.

DBP15: Phenol. Gray powder. The compound was identified by comparison with an authentic, commercially available sample.

DBP16: 1,3-Dihydroxybenzene. Gray powder. The compound was identified by comparison with an authentic, commercially available sample.

DBP17: 2-Ethylhexan-1-ol. Oily liquid. The compound was identified by comparison with an authentic, commercially available sample.

DBP18: 2-Ethylhexanoic acid. Dense liquid. The compound was identified by comparison with an authentic, commercially available sample.

DBP19: Heptane. Oily liquid. The compound was identified through a comparison with an authentic, commercially available sample.

### 2.4. Degradation of Bemotrizinol vs. Ethylhexyltriazone

It is interesting to compare the chemical behaviors of bemotrizinol and tris(2-Ethylhexyl) 4,4′,4″-((1,3,5-triazine-2,4,6-triyl)tris(azanediyl))tribenzoate or, more simply, ethylhexyl triazone (EHT) in relation to the chlorination reaction [37]. EHT is a soluble organic UV-B filter that absorbs UV-B radiation from about 280 to 320 nm.

**Figure 4:**
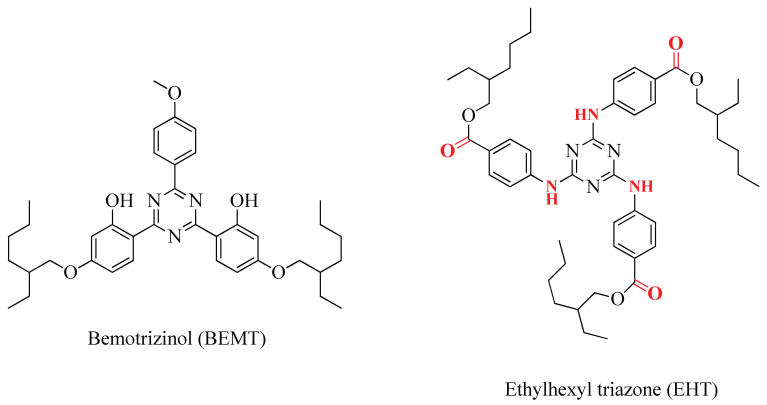
Comparison of the structures of bemotrizinol and ethylhexyl triazone.

Structurally, the two UV filters are very similar, both having a central 1,3,5-triazine ring that is linked to the C-2, C-4, and C-6 carbons directly through three phenyl rings in the case of BEMT and through a bond with NH in the case of EHT (Figure 4). In the case of BEMT, two of the three aromatic rings are para-bonded to a 2-ethylhexyl chain through an O atom, while in the case of EHT, all three aromatic rings are 1,4-disubstituted and have the same 2-ethylhexyl chain para-bonded to the triazine ring but bonded through a carboxylate function.

In the case of bemotrizinol, with a BEMT/NaClO aq. molar ratio of 1:100, the product is recovered in a quantity equal to approximately 2% of the initial amount. Furthermore, 19 DBPs were obtained with an approximate percentage of 51% (46% for DBP1), and mineralization for the remaining 47% (Table 1). With a lower BEMT/NaClO aq. molar ratio of approximately 1:80, the percentage of BEMT recovery increased, equal to approximately 20%, and the percentage of DBP1 formation slightly decreased, settling at around 42%. With an even lower BEMT/NaClO aq. ratio of approximately 1:25, bemotrizinol was recovered up to 91%, accompanied by just over 1% of DBP1.

With an EHT/NaClO aq. ratio of 1:25, EHT recovered as-is at a percentage of 62%. Furthermore, 12 DBPs were isolated in a total quantity equal to 25% of the initial quantity of EHT, and with a mineralization percentage of 13% (Table 1) [37].

When using the same amount of NaClO (BEMT or EHT/NaClO at 1:25), the amount of BEMT recovered was much higher than the corresponding amount of EHT (91 vs. 62%), which indicates that BEMT is more recalcitrant to degradation or mineralized in the reaction with hypochlorite. This was confirmed for EHT by the isolation of 12 DBPs, with a total yield of approximately 25%, compared to only 1 product detected (DBP1) in the case of BEMT, with a yield not exceeding 1%. In the case of BEMT, as the amount of NaClO increased (from 1:20 to 1:80 and up to 1:100), the number of identified DBPs increased (DBP1-DBP19), even though the most abundant was DBP1 (46% of the total transformation percentage, equal to 51%). It is easy to assume that the EHT molecule presents intrinsic breaking points at the NH bridges through which the phenyls are linked to the central triazine ring, and/or at the carboxylate functions through which the 2-ethylhexyl side chains are linked to the phenyls themselves. Fragmentation points were missing in BEMT, which decomposed only in the presence of extremely considerable amounts of NaClO. In general, this suggests that legislative and control authorities could incentivize those who produce active ingredients with the same effects, UV filters in this specific case, to promote the production and use of materials that are easier to degrade and less recalcitrant in wastewater treatment processes, in particular.

## 3. Materials and Methods

### 3.1. Drug and Reagents

Bemotrizinol (≥98%) was purchased from Sigma Aldrich (Milan, Italy). Solvents were purchased from Merck (Darmstadt, Germany), and were of HPLC-grade and used as received. All other chemicals were of analytical grade and supplied by Merck.

### 3.2. Sodium Hypochlorite Reaction

#### 3.2.1. Apparatus and Equipment

Column chromatography (CC) was conducted with Kieselgel 60 (230–400 mesh, Merck, Darmstadt, Germany). HPLC was performed on a Shimadzu LC-8A system using a Shimadzu SPD-10A VP UV-VIS detector (Shimadzu, Milan, Italy). The ^1^H- and ^13^C-NMR spectra were recorded with an NMR spectrometer operated at 400 MHz and at 25 °C (Bruker DRX, Bruker Avance, Billerica, MA, USA) and referenced in ppm to the residual solvent signals (CDCl_3_, at δ_H_ 7.27 and δ_C_ 77.00; CD_3_OD, at δ_H_ 3.31 and δ_C_ 49.15; DMSO-D6, at δ_H_ 2.51 and δ_C_ 39.51). The proton-detected heteronuclear correlations were measured using a gradient heteronuclear single-quantum coherence (HSQC) experiment, optimized for ^1^J_HC_ = 155 Hz, and a gradient heteronuclear multiple bond coherence (HMBC) experiment, optimized for ^n^J_HC_ = 8 Hz. The MALDI-TOF mass spectrometric analyses were performed on a Voyager-De Pro MALDI mass-spectrometer (PerSeptive Biosystems, Framingham, MA, USA). Gas chromatography analyses were obtained on a Shimadzu model GC2010 instrument (Cernusco s/Naviglio, Milan, Italy). The UV/Vis spectra were recorded with a PerkinElmer Lambda 7 spectrophotometer. The IR spectra were recorded with a Jasco FT/IR-430 instrument equipped with a single-reflection ATR accessory, and the samples were dissolved in CHCl_3_ and deposited on a ZnSe crystal.

#### 3.2.2. Analytical and Preparative Experiments

**Figure 5:**
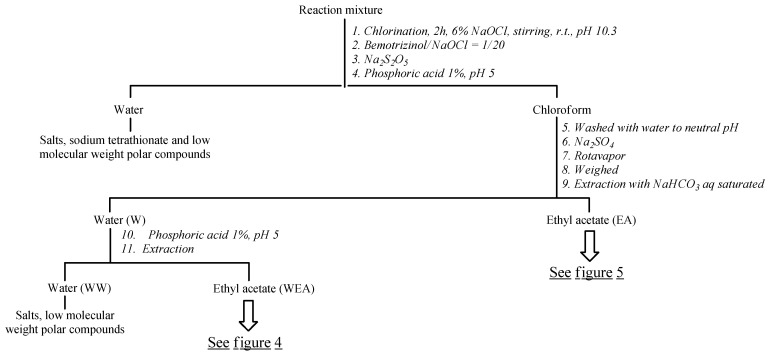
Liquid–liquid extractions of the reaction mixture.

A suspension of BEMT at a concentration of 10^−5^ M was treated with NaClO aq. (a BEMT/NaClO molar ratio of 1:80) for 2 h at a room temperature of 25 °C. BEMT was detected using a HPLC with a UV–Vis detector (Shimadzu, Milan, Italy), quantifying its presence through a calibration curve prepared from solutions of known BEMT concentrations [38]. DBP1–DBP19 (Figure 2) obtained under these conditions were too low in abundance to be isolated. Therefore, it was necessary to compare their retention times with those of the byproducts obtained from BEMT degradation in experiments at a hundred-fold higher concentration, at 10^−3^ M [39,40]. This suspension was treated for 2 h with NaClO aq. (a BEMT/NaClO molar ratio of 1:100) at room temperature. DBPs common to both analytical and quantitative experiments were then isolated from the chloroform extract of the aqueous solution (Figure 5).

#### 3.2.3. Protocol for the Degradation and Isolation of Degradation Byproducts/Preparative Experiment

After 2 h, the chlorination reaction of bemotrizinol at the highest concentration (preparative conditions, 980 mg/L) was stopped by adding sodium thiosulfate (Na_2_S_2_O_3_), and the pH of the suspension was lowered to a value of about 5 by adding 1% phosphoric acid (Figure 5). The obtained suspension was then extracted with chloroform. If the aqueous phase, containing mainly salts, sodium tetrathionate (Na_2_S_4_O_6_), and highly polar and low-molecular-weight compounds, was neglected, then the organic phase was washed with water until neutrality, dehydrated over sodium sulfate (Na_2_SO_4_), dried in a rotavapor, and finally weighed (815 mg). The organic/chloroform phase was redissolved in ethyl acetate and then extracted with a saturated aqueous solution of sodium bicarbonate, obtaining an aqueous phase (W) and an organic phase (EA). The former was adjusted to pH 5 by adding 1% phosphoric acid and then extracted with ethyl acetate. 

**Figure 6:**
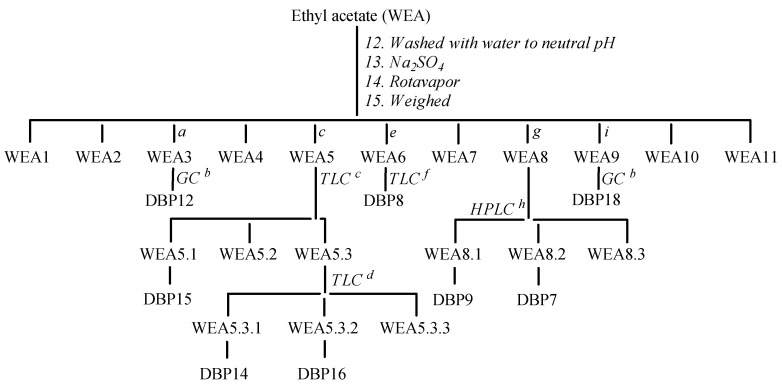
Isolation of DBP7-DBP9, DBP12, DBP14-DBP16, and DBP18. ^a^ Petrol ether-CH_3_COCH_3_ (90:10). ^b^ See experimental part. ^c^ Petrol ether-CH_3_COCH_3_ (80:20). ^d^ CH_2_Cl_2_-CH_3_COCH_3_ (70:30). ^e^ Petrol ether-CH_3_COCH_3_ (70:30). ^f^ CH_2_Cl_2_-CH_3_COCH_3_ (80:20). ^g^ Petrol ether-CH_3_COCH_3_ (50:50). ^h^ CH_3_CN-HCOONH_4_. ^i^ CH_2_Cl_2_-CH_3_COCH_3_ (30:70).

The aqueous phase (WW) contained salts and low-molecular-weight polar compounds, while the organic phase (WEA) was washed with water until it reached a neutral pH, dehydrated over Na_2_SO_4_, dried, and finally weighed (250 mg). The crude reaction was chromatographed with a gradient of petrol ether/CH_3_COCH_3_ (99:1 to 50:50, *v*/*v*) and with CH_2_Cl_2_/CH_3_COCH_3_ (80:20 to 0:100, *v/v*) on silica gel to yield eleven fractions (WEA1-EA11, Figure 6). The WEA3 fraction (10 mg), eluted with petrol ether–CH_3_COCH_3_ (90:10, *v*/*v*), was dried, dissolved in an appropriate volume of CH_3_COCH_3_ (100 μL), and analyzed using a GC-FID instrument. The gas chromatograph was equipped with a Zebron™ ZB-FFAP, GC capillary column (30 m × 0.32 mm × 0.25 μm film thickness, Phenomenex, Bologna, Italy). The following parameters were set during the experiments: 320 °C detector FID temperature, 1.8 mL/min constant flow of helium (carrier gas), 0.6 μL injected sample volume, 250 °C injector temperature, 10:1 split ratio, 40 °C oven profile (held for 4 min), and 5 °C/min to 250 °C (held for 10 min). DBP12 (t_R_ 8 min, 1.6 mg) was identified by comparing the results with data on standard compounds and their relative calibration lines. The WEA5 fraction (51 mg), eluted with petrol ether–CH_3_COCH_3_ (80:20, *v*/*v*), was purified via preparative TLC using the same solvent mixture as above, resulting in three subfractions, of which the first (the WEA5.1 fraction) contained DBP15 (3 mg). The third fraction (WEA5.3, 23 mg) was chromatographed again via semipreparative TLC eluting with CH_2_Cl_2_-CH_3_COCH_3_ (70:30, *v*/*v*), yielding three other fractions, of which the first two, WEA5.3.1 and WEA5.3.2, contained DBP14 (2 mg) and DBP16 (1 mg), respectively. The WEA6 fraction (15 mg), eluted with petrol ether–CH_3_COCH_3_ (70:30, *v*/*v*), was purified via preparative TLC eluting with CH_2_Cl_2_-CH_3_COCH_3_ (80:20, *v*/*v*), yielding DBP8 (2 mg). The WEA8 fraction (22 mg), eluted with petrol ether–CH_3_COCH_3_ (50:50, *v*/*v*), was purified using HPLC, with a column Phenomenex Synergi 10 µm 110 Å C18 (250 × 21.20 mm), and eluted with CH_3_CN and HCOONH_4_, 15 mM, pH 3.75, starting with 95% of HCOONH_4_ for 5 min, followed by the installation of a gradient to obtain 95% of CH_3_CN over 25 min, at a solvent flow rate of 1.5 mL/min. 

Of the three fractions obtained, the first (WEA8.1) contained DBP9 (3 mg) and the second (WEA8.2) contained DBP7 (4 mg). The WEA9 fraction (11 mg), eluted with CH_2_Cl_2_-CH_3_COCH_3_ (30:70, *v*/*v*), was dried, dissolved in an appropriate volume of CH_3_COCH_3_ (100 μL), and analyzed using a GC-FID instrument. The gas chromatograph was equipped with an Equity™-5 GC capillary column (30 m × 0.25 mm × 0.25 μm film thickness, Supelco, Sigma-Aldrich Group, St. Louis, MO, USA). The following parameters were set during the experiments: 340 °C detector FID temperature, 25 cm/sec constant flow of helium (carrier gas), 1.0 μL injected sample volume, 225 °C injector temperature, 50:1 split ratio, 40 °C oven profile (held for 5 min), and 8 °C/min to 300 °C (held for 10 min). DBP18 (t_R_ 11 min, 1.4 mg) was identified by comparing the result with data on standard compounds and their relative calibration lines.

**Figure 7:**
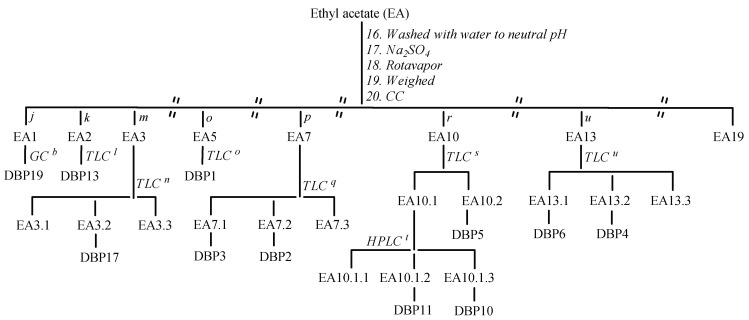
Isolation of DBP1-DBP6, DBP10-DBP11, DBP13, DBP17, and DBP19. ^b^ See experimental part. ^j^ Petrol ether-CH_3_COCH_3_ (99:1). ^k^ Petrol ether-CH_3_COCH_3_ (97:3). ^l^ Petrol ether-CH_3_COCH_3_ (95:5). ^m^ Petrol ether-CH_3_COCH_3_ (92:8). ^n^ CH_2_Cl_2_-CH_3_COOCH_2_CH_3_ (85:15). ^o^ Petrol ether-CH3COCH3 (85:15). ^p^ Petrol ether-CH_3_COCH_3_ (80:20). ^q^ CH_2_Cl_2_-CH_3_COOCH_2_CH_3_ (75:25). ^r^ Petrol ether-CH_3_COCH_3_ (75:25). ^s^ CH_2_Cl_2_-CH_3_COCH_3_ (85:15). ^t^ CH_3_CN-HCOOH 0.1% (50:50 to 100:0). ^u^ CH_2_Cl_2_-CH_3_COCH_3_ (60:40).

The organic fraction EA (Figure 7) was washed with water until neutral pH, dehydrated over Na_2_SO_4_, dried, and finally weighed (565 mg). The reaction crude was chromatographed with a gradient of petrol ether/CH_3_COCH_3_ (99:1 to 60:40, *v*/*v*) and with CH_2_Cl_2_-CH_3_COCH_3_ (60:40, *v*/*v*) on silica gel to yield 19 fractions (EA1-EA19). The EA1 fraction (7 mg), eluted with petrol ether–CH_3_COCH_3_ (99:1, *v*/*v*), was dried, dissolved in an appropriate volume of CH_3_COCH_3_ (100 μL), and analyzed using a GC-FID instrument. The gas chromatograph was equipped with a Petrocol DH 50.2 column (50 m × 0.20 mm × 0.50 μm film thickness, Supelco, Sigma-Aldrich Group, St. Louis, MO, USA). The following parameters were set during the experiments: 260 °C detector FID temperature, 20 cm/sec constant flow of helium (carrier gas), 1.0 μL injected sample volume, 200 °C injector temperature, 100:1 split ratio, 35 °C oven profile (held for 25 min), and 2 °C/min to 200 °C (held for 15 min). DBP19 (t_R_ 11 min, 2.2 mg) was identified by comparing the result with data on standard compounds and their relative calibration lines. The EA2 fraction (16 mg), eluted with petrol ether–CH_3_COCH_3_ (97:3, *v*/*v*), was purified via preparative TLC under condition *l*, entailing petrol ether–CH_3_COCH_3_ (95:5, *v*/*v*), yielding DBP13 (1 mg). The EA3 fraction (21 mg), eluted with petrol ether–CH_3_COCH_3_ (92:8, *v*/*v*), was purified via preparative TLC under condition *n*, entailing CH_2_Cl_2_-CH_3_COOCH_2_CH_3_ (85:15, *v*/*v*), resulting in three subfractions, of which the second (EA3.2) contained the product DBP17 (13 mg). The EA5 fraction (11 mg), eluted with petrol ether–CH_3_COCH_3_ (85:15, *v*/*v*), was purified via preparative TLC eluting with the same solvent mixture, yielding DBP1 (4 mg). The EA7 fraction (18 mg), eluted with petrol ether–CH_3_COCH_3_ (80:20, *v*/*v*), was purified via preparative TLC under condition *q*, entailing CH_2_Cl_2_-CH_3_COOCH_2_CH_3_ (75:25, *v*/*v*), resulting in three subfractions, of which the first (the EA7.1 fraction) contained DBP3 (2 mg) and the second (the EA7.2 fraction) contained DBP2 (2 mg). The EA10 fraction (27 mg), eluted with petrol ether–CH_3_COCH_3_ (75:25, *v*/*v*), was purified via preparative TLC under condition *s*, entailing CH_2_Cl_2_-CH_3_COCH_3_ (85:15, *v*/*v*), which resulted in two subfractions, of which the second (the EA10.2 fraction) contained DBP5 (2 mg). Instead, the first fraction (EA10.1, 22 mg) was purified using HPLC with a column Phenomenex Synergi 10 µm 110 Å C18 (250 × 21.20 mm) and was eluted with CH_3_CN and HCOOH 0.1%, starting with 50% of CH_3_CN for 3 min, followed by the installation of a gradient to obtain 100% of CH_3_CN over 20 min, at a solvent flow rate of 1.2 mL/min. Of the three fractions obtained, the second (EA10.1.2) contained DBP11 (2 mg) and the third (EA10.1.3) contained DBP10 (1 mg). The EA13 fraction (23 mg), eluted with CH_2_Cl_2_-CH_3_COCH_3_ (60:40, *v*/*v*), was purified via preparative TLC eluting with the same solvent mixture, resulting in three subfractions, of which the first (the EA13.1 fraction) contained DBP6 (3 mg) and the second (the EA13.2 fraction) contained DBP4 (2 mg).

## 4. Conclusions

Bemotrizinol is a UV filter that is used not only in many personal care products, such as sunscreen and cosmetics, but also as an additive in plastics or clothing to protect against damage that results from absorbed radiation. It is an emerging pollutant and, not surprisingly, it has been detected in wastewater, surface water, and some lake sediments.

In this study, the UV filter was subjected to oxidation with sodium hypochlorite, which is the most used oxidant in wastewater treatment plants or in swimming pool disinfection processes. Nineteen degradation byproducts were obtained, purified using different chromatographic methods (CC, TLC, HPLC, and GC), and identified using one- and two-dimensional nuclear magnetic resonance (NMR) spectroscopy and mass spectrometry. Finally, a plausible reaction mechanism was proposed to explain how all the identified byproducts were obtained. Of the isolated DBPs, only three retained the central nucleus consisting of a 1,3,5-triazine ring. The other 16 derived from the degradation of the phenyl-type aromatic rings, linked to the central nucleus and their side chains. It is interesting to note that ethylhexyl triazone, a sunscreen with a very similar structure but with phenyl rings linked to the central nucleus by nitrogen bridges, was recovered at an unchanged percentage of 62%, compared to the 91% found for bemotrizinol with the same concentration of oxidant used. Evidently, the former is much less recalcitrant than the latter.

## Figures and Tables

**Table 1:** Recovery, transformation, and mineralization rates of BEMT and EHT and the number of DBPs identified.

	% BEMT	No. DBPs	%DBPs	%DBP1	Mineralization
BEMT–NaClO aq. di 1:100	2	19	51	46	47
BEMT–NaClO aq. di 1:80	20			42	
BEMT–NaClO aq. di 1:25	91			1	
	% EHT	Num. DBPs	%DBPs		Mineralization
EHT–NaClO aq. di 1:25	62	12	25		13

## Data Availability

The original contributions presented in this study are included in the article. Further inquiries can be directed to the corresponding author.
